# Supplementary material for: Heterogenous Genetic, Clinical, and Imaging Features in Patients with Neuronal Intranuclear Inclusion Disease Carrying NOTCH2NLC Repeat Expansion
Source: Brain Sci. 2023 Jun 15;13(6):955. doi: 10.3390/brainsci13060955 (PMC10296744; doi:10.3390/brainsci13060955)
Supplement: Supplementary file 1 [file brainsci-13-00955-s001.zip › brainsci-2433354-supplementary.pdf]

Supplementary Table 1. Association between expanded *NOTCH2NLC* repeat size and clinical/MRI features

	Expanded *NOTCH2NLC* total repeat number, mean ± SD (range)		*P* value
	present	absent	
**Clinical symptoms**			
Cognitive decline	148.5 ± 78.3 (97–361, n = 11)	111.0 ± 20.7 (94–141, n = 4)	0.32
Hyporeflexia	151.7 ± 80.9 (97–361, n = 10)	112.2 ± 26.0 (94–361, n = 4)	0.12
Consciousness disturbance	145.0 ± 84.9 (94–361, n = 9)	129.2 ± 40.2 (97–198, n = 6)	0.98
Cerebellar ataxia	133.0 ± 36.7 (97–198, n = 9)	146.8 ± 105.2 (94–361, n = 6)	0.37
Dysarthria	124.3 ± 29.7 (97–175, n = 8)	154.9 ± 97.5 (94–361, n = 7)	0.89
Encephalitis episodes	145.3 ± 90.9 (94–361, n = 8)	130.9 ± 37.0 (97–198, n = 7)	0.67
Gait disturbance	132.0 ± 39.1 (97–198, n = 8)	146.0 ± 96.1 (94–361, n =7)	0.59
Tremor	107.6 ± 5.9 (101–117, n = 7)	165.6 ± 87.8 (94–361, n = 8)	0.41
Urinary dysfunction	141.4 ± 37.8 (97–198, n = 7)	136.0 ± 91.1 (94–361, n = 8)	0.18
Psychiatric symptoms	162.9 ± 94.6 (97–361, n = 7)	117.3 ± 27.5 (94–175, n = 8)	0.27
**MRI features**			
Cerebellar atrophy	141.7 ± 80.4 (94–361, n = 11）	129.8 ± 24.0 (104–158, n = 4)	0.39
DWI high-intensity lesions in corpus callosum	147.7 ± 82.4 (97–361, n = 10)	120.2 ± 27.8 (94–158, n = 5)	0.53
DWI high-intensity lesions in posterior lobe	134.0 ± 41.7 (101–198, n = 6)	141.6 ± 85.1 (94–361, n = 9)	0.47
Paravermal lesions	104.0 ± 7.7 (97–117, n = 5)	155.8 ± 80.0 (94–361, n = 10)	0.07

FLAIR, fluid attenuated inversion recovery; DWI, diffusion weighted imaging

Supplementary Figure 1

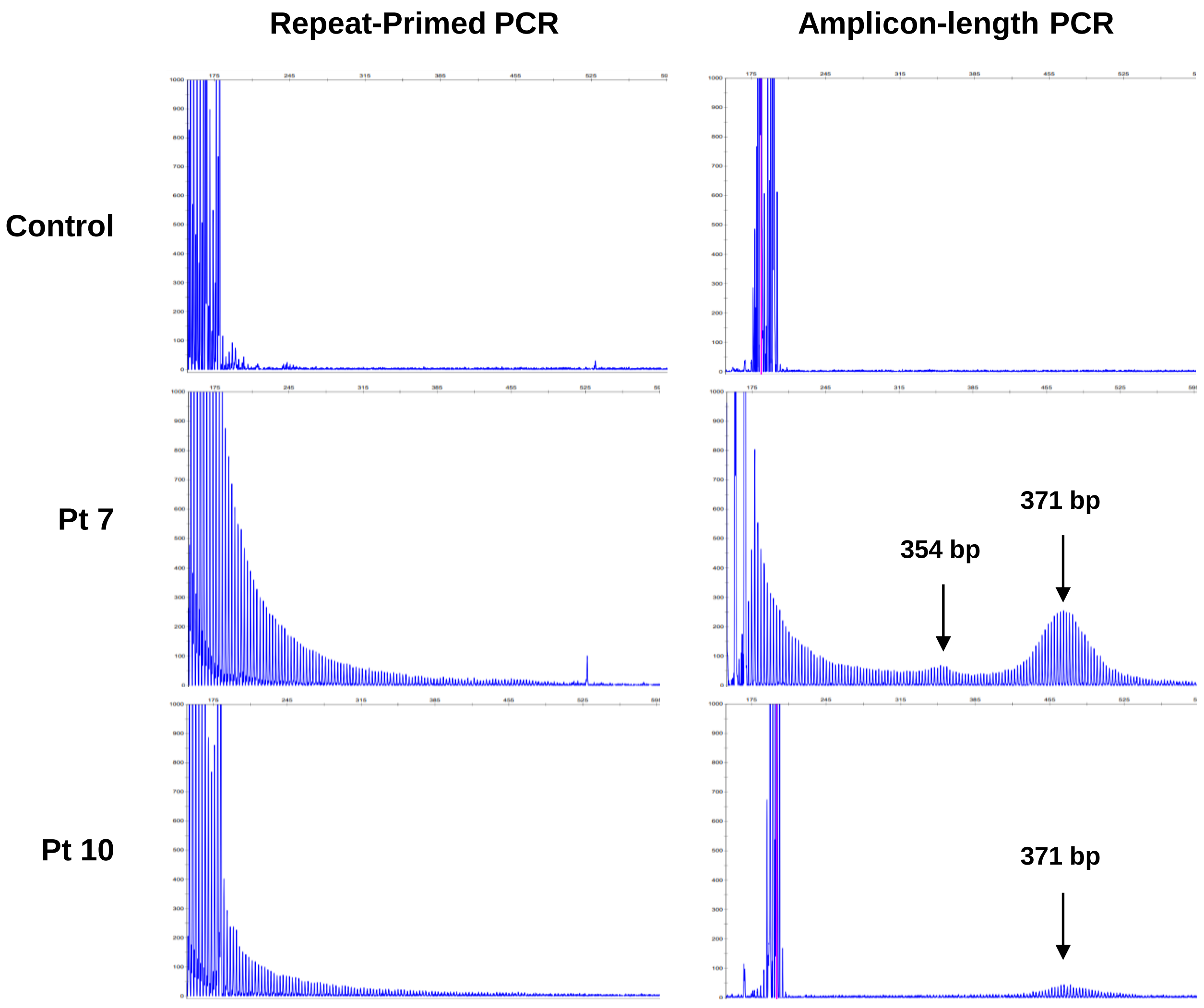

Figure Legend

Representative results of repeat-primed and amplicon-length PCR of *NOTCH2NLC*. In Pt 7 and 10, a sawtooth pattern was observed by repat-primed PCR (*left*). In Pt 7, two peaks at 354 and 371 bp, corresponding to expanded alleles, were observed by amplicon-length PCR (*right*). In Pt 10, a single peak at 371 bp, corresponding to an expanded allele, was observed.

Supplementary Figure 2

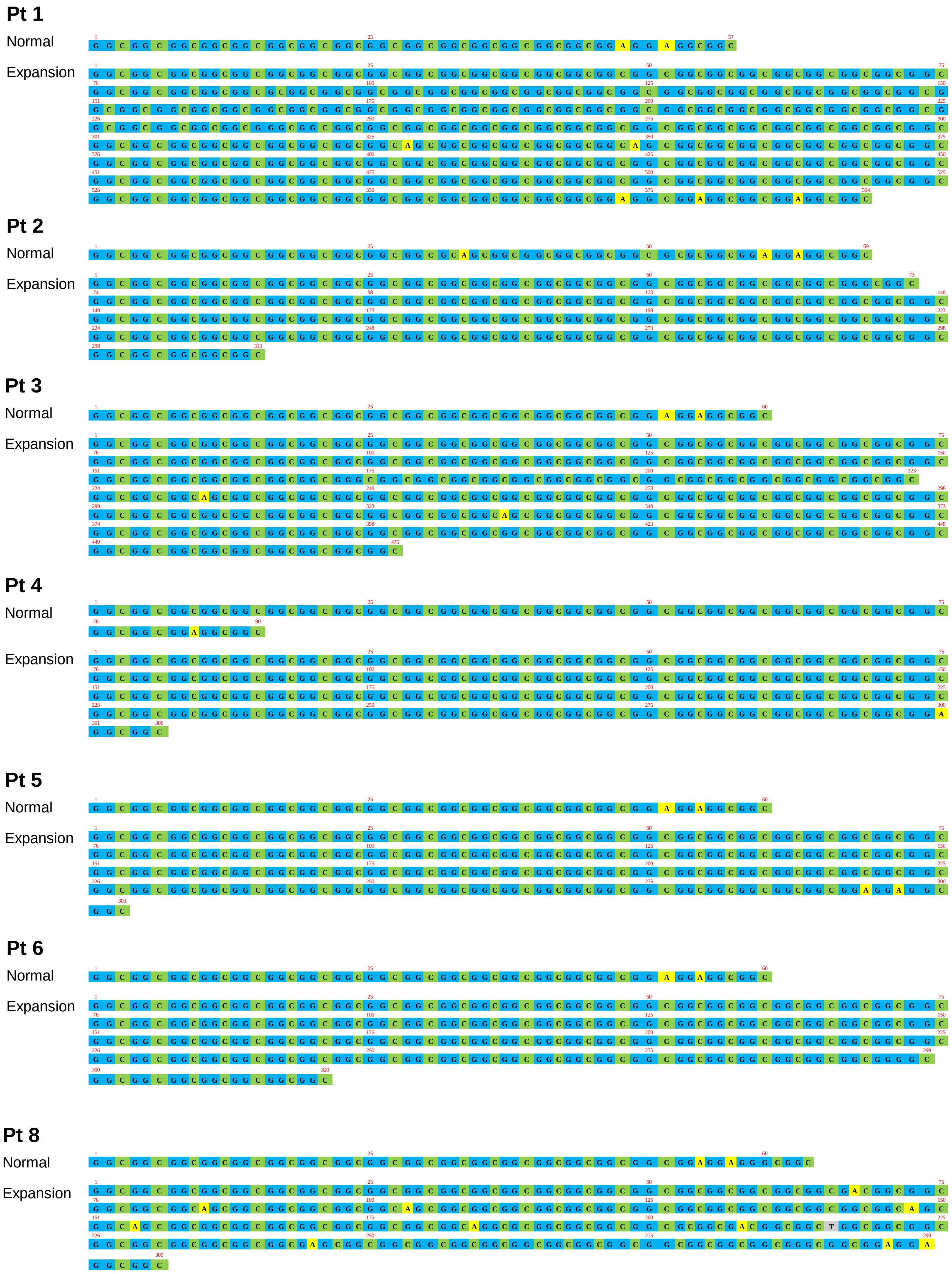

Supplementary Figure 2 (continued)

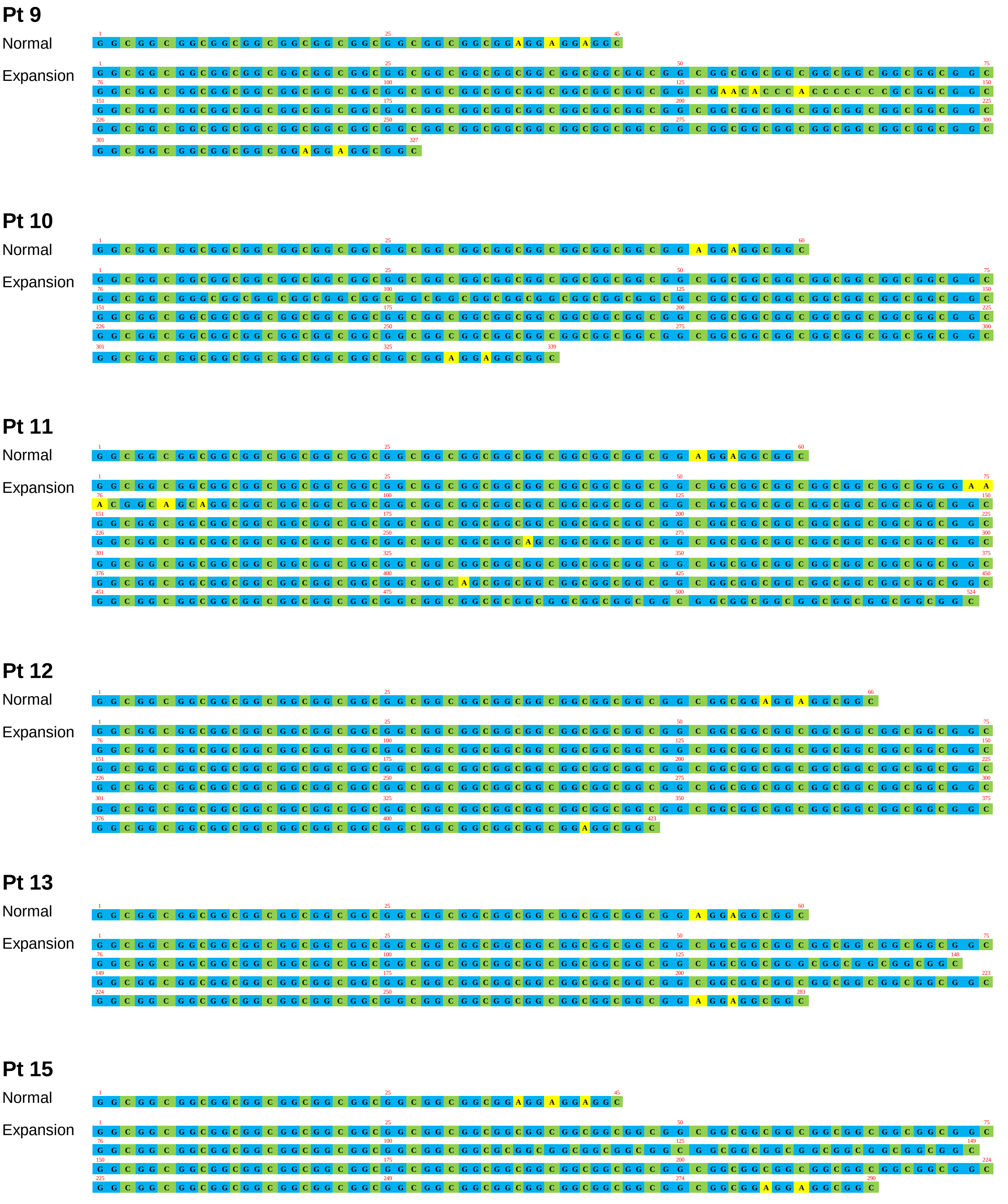

Figure Legend

Repeat sequences in *NOTCH2NLC* in patients with NIID were shown. Pt 2 carried a pure GGC repeat expansion. Other patients carried GGC repeat expansions with other trinucleotide sequences such as GGA, AGC, and GAC.

Supplementary figure 3

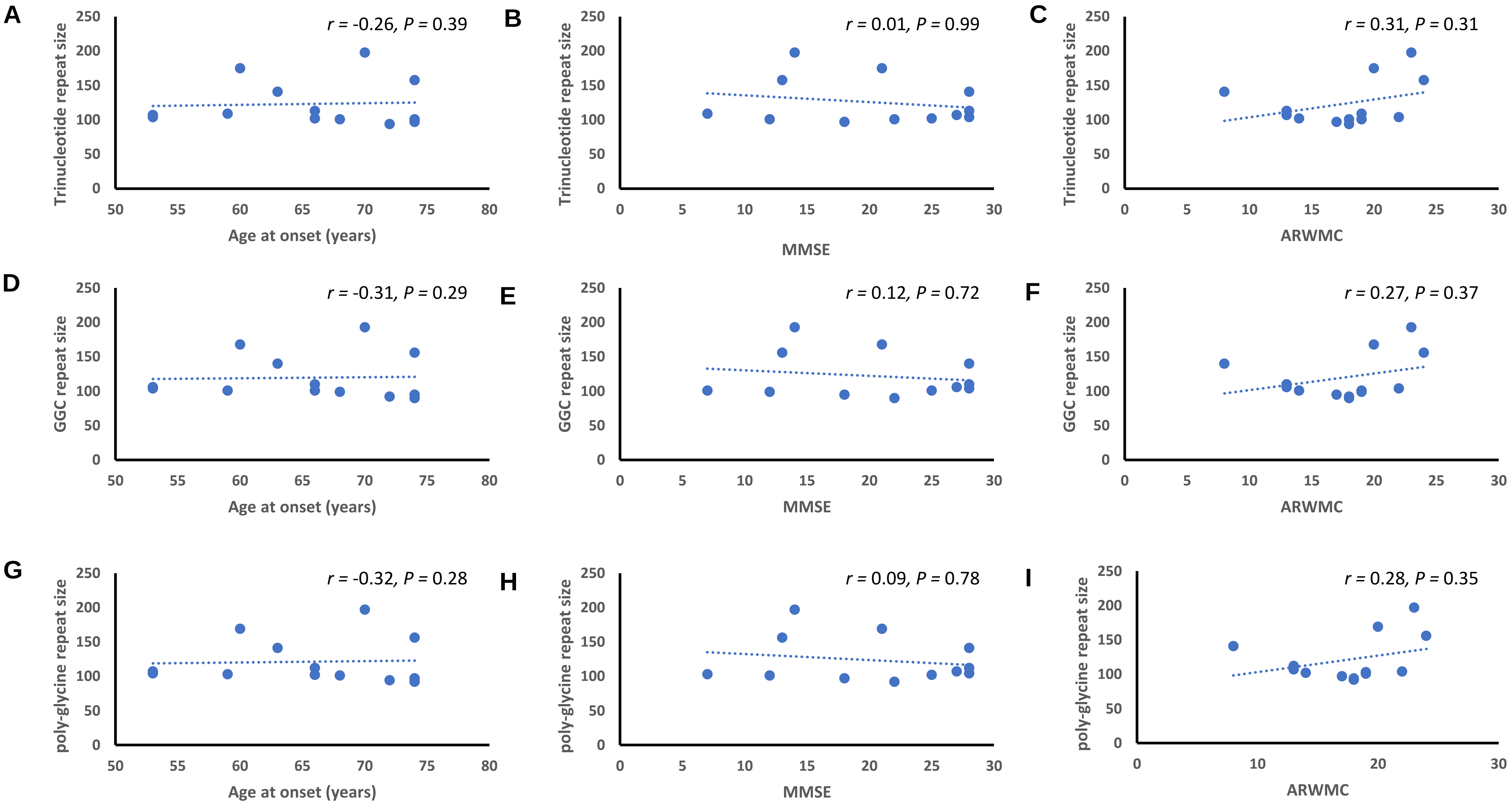

Figure legend

Correlation between trinucleotide repeat length and age at onset **(A)**, MMSE **(B)**, and ARWMC **(C)** were shown. Correlations of these parameters were analyzed based on cumulative GGC repeat length **(D-F)** or poly-glycine repeat length **(G-I)**.
